# Screening, assessment, and treatment of osteoporosis for the nurse practitioner: Key questions and answers for clinical practice—A Canadian perspective

**DOI:** 10.1002/2327-6924.12134

**Published:** 2014-06-09

**Authors:** Peggy Rice, Upender Mehan, Celeste Hamilton, Sandra Kim

**Affiliations:** 1 Lakeridge Health Whitby PASS ProgramWhitby, Ontario, Canada; 2 Department of Family Medicine, McMaster UniversityHamilton, Ontario, Canada; 3 Department of Family Medicine, Western UniversityLondon, Ontario, Canada; 4 Department of Scientific Affairs, Amgen Canada IncMississauga, Ontario, Canada; 5 Department of Medicine, Division of Endocrinology & Metabolism, Women's College HospitalToronto, Ontario, Canada; 6 Department of Medicine, University of TorontoToronto, Ontario

**Keywords:** Osteoporosis, clinical practice guidelines, bone health, screening, assessment, treatment, nurse practitioner

## Abstract

**Purpose:**

Using a case-based approach, we review key clinical questions relevant to nurse practitioners (NPs) regarding the screening, assessment, and treatment of patients at risk for osteoporosis and fractures in a Canadian general practice setting.

**Data sources:**

A case presentation with relevant questions and answers to guide management of a patient.

**Conclusions:**

Osteoporosis is a common condition in both the aging male and female populations. Screening, diagnosis, and treatment of osteoporosis is lagging behind relative to other chronic disease states. NPs have a unique opportunity to help reduce this care gap by playing an integral role in the identification, risk stratification, and treatment of patients at risk for osteoporosis and fractures.

**Implications for practice:**

This case highlights the important role an NP can have in screening a patient previously not diagnosed or managed for osteoporosis. Performing a focused history and physical exam of the patient to determine appropriate screening tests and fracture risk will help in guiding treatment decisions.

## Overview

Fragility fractures as a result of osteoporosis in both men and women represent a major health concern. The Canadian Multicentre Osteoporosis Study (CaMos) showed that the prevalence of osteoporosis in men and women was 21.5% and 23.5%, respectively (Jackson, Tenenhouse, Robertson, & CaMos Study Group, 2000), with a corresponding fracture incidence of 7.6% and 12.5% (Ioannidis et al., 2009) after the age of 50 years. Of note, more than 80% of the fractures in women after the age of 50 are fragility fractures (Bessette et al., 2008), and approximately 40% of individuals that suffer a fragility fracture will suffer another fracture within a year (Hajcsar, Hawker, & Bogoch, 2000).

The impact of a fragility fracture on a patient's quality of life is notable, with approximately 40% of women over the age of 50, who have had a hip fracture requiring assistance with ambulation 1 year after the fracture (Cooper, 1997), and 18% requiring long-term care following hospitalization (Jean et al., 2013). Fractures have both morbidity and mortality consequences. In CaMos, both men and women showed an increased incidence of death following a hip fracture of 23.5% (Ioannidis et al., 2009). Furthermore, the impact of a fragility fracture not only affects the individual's quality of life, but often has negative consequences on the family and social network, work productivity, income potential, and healthcare system. The economic burden of osteoporosis in Canada is large with annual costs of hip fracture estimated at $13,111 for men and $16,171 for women (Leslie, Metge et al., 2011).

Despite this evident clinical and economic burden, only 2.3% of men (Papaioannou et al., 2008) and 20% of women (Hajcsar et al., 2000) in Canada are investigated for osteoporosis following a fragility fracture. Moreover, less than 10% of men (Papaioannou et al., 2008) and 20% of women (Bessette et al., 2008; Papaioannou et al., 2004) are treated with osteoporosis medication after sustaining a fragility fracture, demonstrating a care gap in the diagnosis, management, and treatment of osteoporosis by healthcare providers.

If one compares osteoporosis to the inroads made in the management of patients following an acute myocardial infarction (MI), it is possible that such a care gap can be overcome. In the early 1990s after an MI, approximately 42% of patients filled a prescription for a beta blocker and a statin. By 2005, almost 80% of the patients filled prescriptions for these two medications (Austin, Tu, Ko, & Alter, 2008) to prevent a future myocardial event. In this area of cardiology, evidence-based medicine provided the necessary information to highlight the impact of medications on the secondary prevention of coronary events. In osteoporosis, evidence exists with a number of medications, that appropriate treatment can effectively reduce the risk of future fragility fractures. The key to addressing the care gap then, is to ensure appropriate identification, diagnosis, and treatment of patients at risk for fracture.

Nurse practitioners (NPs) are an important adjunct to physicians in the diagnosis, management, and follow-up of patients with numerous diseases including osteoporosis. There are now more than 3000 NPs in Canada, with every province and territory having legislation in place surrounding NPs (http://www.npcanada.ca). In the past, the role of NPs in osteoporosis management was focused on counseling efforts regarding osteoporosis prevention and lifestyle modifications. However, in recent years, and with the ability to prescribe medications, the NP's role has evolved and includes involvement in all aspects of osteoporosis management.

The purpose of this article is to use a case-based approach to review key clinical questions and answers relevant to NPs regarding the screening, assessment, and treatment of osteoporosis in a Canadian general practice setting.

## Case study

A 69-year-old female patient Mrs. X, visits her NP for her annual healthcare visit. She has a body mass index (BMI) of 27 kg/m^2^ and is able to carry out normal daily activities.

### Screening

Question: Who should be assessed for osteoporosis and fracture risk?

*Answer*: Both men and women over the age of 50 years should be assessed for fracture risk as well as any individual who has experienced a prior fragility fracture (Papaioannou et al., 2010). A fragility fracture is a fracture that occurs with minimal or no trauma such as a fall from a standing height or height less than 1 m, and includes all bones except the skull and face, patella, hand or finger, toe, cervical spine, and metatarsus (Bessette et al., 2008).

Question: What do I need to consider when screening patients for osteoporosis and fracture risk?

*Answer*: When screening for osteoporosis, a detailed history should be conducted along with a focused physical examination to identify risk factors for low bone mass, fractures, and falls, as well as undiagnosed vertebral fractures (Papaioannou et al., 2010). Table 1 summarizes patient history areas to focus on, as well as physical exam maneuvers to identify risk factors for fracture. The assessment should also include questions about dietary and supplementation intake of calcium and vitamin D.

**Table 1:** Clinical assessment of an individual at risk of osteoporosis^a^

Assessment	Identify risk factors for low BMD, fractures, and falls
History	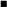 Prior fragility fractures
	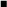 Parental hip fracture
	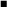 Glucocorticoid use
	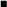 Current smoking
	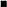 High alcohol intake (≥3 units/day)
	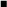 Rheumatoid arthritis
	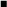 Inquire about falls in the previous 12 months
	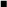 Inquire about gait and balance
	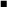 Other comorbidities and medications associated with osteoporosis and fractures
Physical examination	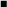 Measure weight (weight loss of >10% since age 25 is significant)
	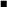 Measure height annually (prospective loss > 2 cm; historical height loss >6 cm)^b^
	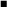 Measure rib to pelvis distance ≤2 fingers breadth^b^
	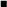 Measure occiput-to-wall distance (for kyphosis) > 5 cm^b^
	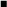 Assess fall risk by using get-up-and-go test (ability to get out of chair without using arms, walk several steps, and return)

a Adapted from Papaioannou et al.'s (2010) Clinical Practice Guidelines Osteoporosis: Background and Technical Report (http://www.osteoporosis.ca/multimedia/pdf/Osteoporosis_Guidelines_2010_Background_And_Technical_Report.pdf).

b Screening for vertebral fractures.

Question: When should I order a bone mineral density (BMD)?

*Answer*: A baseline BMD test should be ordered in all individuals who are 65 years of age or older, and in both men and postmenopausal women between the ages of 50 and 64 with one or more clinical risk factors for bone loss or fracture (Papaioannou et al., 2010; see Table 2). For individuals <50 years of age, specific indications including secondary causes of osteoporosis, prior fragility fracture, or use of high-risk medications may suggest the need for a BMD test (for further details please refer to Table 2).

**Table 2:** Indications for measuring bone mineral density^c^

Adults (age ≥ 65 years)	Adults (age ≥ 50–64 years)	Adults (age < 50 years)
Age ≥ 65 years	Clinical risk factors for fracture (postmenopausal women, men age 50–64 years)	Fragility fracture Prolonged use of glucocorticoids^a^
	•Fragility fracture after age 40 years	Use of other high-risk medications^b^
	•Prolonged use of glucocorticoids^a^	Hypogonadism
	•Use of other high-risk medications^b^	Malabsorption syndrome
	•Parental hip fractures	Primary hyperparathyroidism
	•Vertebral fracture or osteopenia identified on radiography •Current smoking	Other disorders strongly associated with rapid bone loss and/or fracture
	•High alcohol intake	
	•Low body weight (<60 kg) or major weight loss (>10% of body weight at age 25 years)	
	•Rheumatoid arthritis	
	•Other disorders strongly associated with osteoporosis	

1 At least 3 months cumulative therapy in the previous year at a prednisone-equivalent dose ≥7.5 mg daily.

2 For example, aromatase inhibitors or androgen deprivation therapy.

3 Adapted from Papaioannou et al. (2010).

**NP tip**

All patients who are 50 years of age and older should be screened for osteoporosis by reviewing clinical risk factors that are associated with fracture risk based on a thorough medical and medication history, family history, lifestyle choices, and history of prior falls. In addition, a physical exam should be conducted to assess findings suggestive of occult vertebral fracture and fall risk.

#### Case progression

Mrs. X is 69 years of age so she should be screened for osteoporosis. A detailed clinical history reveals neither prior fragility fracture nor family history of parental hip fracture or osteoporosis. She has never used glucocorticoids and does not smoke nor drink excess alcohol. Mrs. X discloses that she has gastroesophageal reflux disease (GERD) that is currently controlled by use of a proton-pump inhibitor for over 2 years, but takes no other prescription medications. She takes a multivitamin for women 50+ as a supplement. Upon assessing Mrs. X's height, you note that she has lost 2.0 cm (approx. 1 inch) compared to 2 years ago. Upon further questioning about falls, she reveals she has fallen twice in the last month. BMD assessment indicates a lumbar spine *T*-score of −1.7 and femoral neck *T*-score of −2.5.

Question: What further investigations should be considered?

*Answer*: Patients with a history of fragility fracture or diagnosis of osteoporosis (*T*-score ≤ −2.5) on BMD, should be further assessed with biochemical testing to rule out secondary causes of osteoporosis as outlined in Tables 3 and 4. In addition, a lateral thoracic and lumbar x-ray (T4 to L4) should be performed on select patients based on positive physical findings as per Table 1 to screen for occult morphometric vertebral fractures.

**Table 3:** Recommended biochemical tests^a^

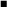 Calcium, corrected for albumin
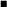 Complete blood count
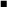 Creatinine
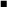 Alkaline phosphatase
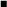 Thyroid-stimulating hormone
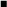 Serum protein electrophoresis (for patients with vertebral fractures)
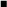 25-Hydroxyvitamin D^b^

a Adapted from Papaioannou et al.'s (2010) Clinical Practice Guidelines Osteoporosis: Background and Technical Report (http://www.osteoporosis.ca/multimedia/pdf/Osteoporosis_Guidelines_2010_Background_And_Technical_Report.pdf).

b Should be measured after 3–4 months of adequate supplementation and should not be repeated if an optimal level (at least 75 nmol/L) is achieved.

**Table 4:** Additional biochemical tests if indicated by clinical assessment^a^

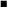 Hyperparathyroidism—if persistently elevated serum calcium	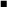 PTH
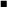 Multiple myeloma—in patients with multiple or atypical vertebral fractures	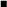 Protein electrophoresis
	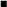 Immunoelectrophoresis
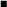 Celiac disease—if symptoms/signs of malabsorption or nonresponse to vitamin D therapy	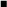 Antibodies associated with gluten enteropathy
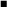 Hypogonadism—in men with signs and symptoms of androgen deficiency	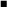 Testosterone (free and total)
	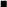 Serum prolactin
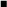 Hypercalciuria—consider in patients with history of kidney stones or high-dose glucocorticoids for prolonged periods	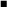 24-hour urine for calcium

a Adapted from Papaioannou et al.'s (2010) Clinical Practice Guidelines Osteoporosis: Background and Technical Report (http://www.osteoporosis.ca/multimedia/pdf/Osteoporosis_Guidelines_2010_Background_And_Technical_Report.pdf).

#### Case progression

Mrs. X had documented height loss, so she was sent for a lateral thoracolumbar spine x-ray, specifying “rule out vertebral compression fracture” on the requisition. Mrs. X was also sent for biochemical testing based on her osteoporotic *T*-score. No vertebral fractures were found and laboratory investigations were normal.

## Risk assessment

Question: How do I assess absolute 10-year fracture risk?

*Answer*: In Canada, two tools are available to determine 10-year risk for a major osteoporotic fracture—(1) the Canadian Association of Radiologists and Osteoporosis Canada (CAROC; Leslie, Berger et al., 2011) and (2) fracture risk assessment (FRAX; Leslie, Lix et al., 2011) tools. The latter is a World Health Organization (WHO) tool, which offers country-specific data. Both tools use age, sex, and BMD *T*-score of the femoral neck to determine fracture risk and should be used in treatment-naïve patients. As well, both tools are calibrated and validated using the same Canadian fracture data.

**Figure 1:**
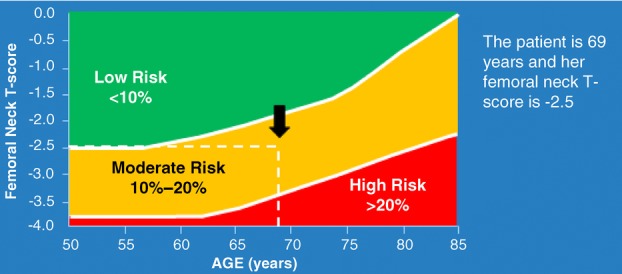
Fracture risk assessment using the CAROC tool for Mrs. X. *Note*. Adapted from Papaioannou et al. (2010).

The CAROC tool stratifies men and women into three zones of risk for a major fracture in the next 10 years, mild (<10%), moderate (10%–20%), and high (>20%). An initial risk category is determined using age, sex, and femoral neck *T*-score (Figure 1). Specific clinical risk factors can further increase a patient's risk to the next risk level independent of BMD, the most important ones being the presence of a prior fragility fracture after age 40 and recent prolonged use of systemic glucocorticoids (i.e., at least 3 months cumulative use within the past year at a prednisone-equivalent dose ≥7.5 mg daily). The presence of both of these risk factors puts the patient in the high-risk category. In addition, patients are automatically considered high risk for future fracture if there is a history of a vertebral fracture or hip fracture, or multiple fragility fractures regardless of BMD results. Although only the femoral neck *T*-score is used to determine the initial risk category in the CAROC tool, when either the lumbar spine or total hip *T*-score is ≤ −2.5, patients should be considered at least moderate risk for fracture (Papaioannou et al., 2010). An online calculator is available from Osteoporosis Canada for the CAROC tool and can be accessed at the following url (http://www.osteoporosis.ca/multimedia/FractureRiskTool/index.html#/Home). A downloadable app for the iPhone and Windows 7 phones can be obtained at http://www.osteoporosis.ca/health-care-professionals/clinical-tools-and-resources/fracture-risk-tool/.

The FRAX tool uses the same information as CAROC, but takes into account additional clinical risk factors including low body mass index, parental hip fracture, current smoking, alcohol intake (≥3 units/day), rheumatoid arthritis, or other secondary causes of osteoporosis (Leslie, Lix et al., 2011). One difference is the optional use of BMD data in the FRAX tool, which may be particularly helpful when BMD scans are not readily available. Although the FRAX tool (http://www.shef.ac.uk/FRAX/tool.aspx?country=19) uses a complete set of clinical risk factors, there is high concordance of 90% between the two tools (Leslie, Berger et al., 2011; Papaioannou et al., 2010). Both tools are recognized in the 2010 Canadian Osteoporosis guidelines (Papaioannou et al., 2010). The choice of tools is therefore a matter of preference and convenience.

**NP tip**

Assess the 10-year fracture risk using either the CAROC or FRAX tool to guide management.

### Case progression

Using either the FRAX or CAROC tool, Mrs. X is estimated to have a moderate 10-year risk (10%–20%) for major osteoporotic fracture.

What are the implications for a patient who is at moderate risk for fracture?

*Answer*: The estimated risk for a patient at moderate risk for fracture is not trivial. An individual approach should be taken to rule out any additional risk factors that may increase the risk level further (e.g., the presence of a vertebral compression fracture would automatically move Mrs. X to the high-risk category). This is important as risk levels help guide management. Specific considerations will be discussed in the treatment initiation section below.

What if the lateral spine x-ray showed compression fractures with a vertebral height loss of >25%? How would this alter her fracture risk?

*Answer*: The presence of a vertebral compression fracture would automatically move Mrs. X to the high-risk category.

### Treatment initiation

Question: Who should be treated with osteoporosis medications?

*Answer*: In general, patients at low risk for fracture are not likely to benefit from pharmacologic treatment but optimization of lifestyle factors including regular exercise, fall prevention, optimal calcium and vitamin D intake, and smoking cessation is recommended. For patients at high risk, good evidence exists showing benefit from pharmacotherapy and therefore is strongly recommended. Patients at moderate risk may benefit from pharmacotherapy depending on the presence of additional risk factors. Risk factors that warrant consideration of pharmacotherapy in the moderate risk patient include vertebral fracture on lateral spine x-ray, wrist fracture in patients older than 65, and those with *T*-score < −2.5, lumbar spine *T*-score much lower than femoral neck *T*-score (by ≥1 SD), rapid bone loss (significant rate of decline in BMD since last scan), chronic systemic glucocorticoids not meeting conventional criteria for recent prolonged use, concurrent high-risk conditions or medications (see Table 5), and recurrent falls with ≥2 falls in the past year (Papaioannou et al., 2010). The benefits versus risks of medication therapy as well as patient preference should be considered in treatment decision making.

**Table 5:** Medications that may increase bone loss or fracture risk

Anticonvulsants^a^
Antipsychotic drugs^b^
Aromatase inhibitors^c^
Chemotherapeutic^d^/transplant drugs (e.g., glucocorticoids)^c^
Furosemide^e^
Hormonal/endocrine therapies—(GnRH agonists, LHRH analogs)^c^
Proton-pump inhibitors^f^
Selective serotonin reuptake inhibitors^g^

*Note*.

LHRH, luteinizing-hormone-releasing hormone.

a Lee, Lyles, and Colón-Emeric (2010).

b Crews and Howes (2012).

c Papaioannou et al. (2010).

d Mayer (2013).

e Drinka, Krause, Nest, and Goodman (2007).

f Targownik et al. (2008).

g Wu, Bencaz, Hentz, and Crowell (2012).

Question: What are the choices of osteoporosis medication?

*Answer*: Regardless of the medication choice, an essential part of the treatment plan is to ensure an adequate intake of calcium and vitamin D. Recommended elemental calcium intake for most individuals over age 50 years is 1200 mg daily and for vitamin D 800–2000 units (20–50 μg) daily achieved from diet and supplements combined (Papaioannou et al., 2010). In addition, regular weight-bearing exercise and fall prevention strategies are key elements of bone health and fracture prevention (Papaioannou et al., 2010).

Pharmacologic therapy has been shown to reduce the risk of vertebral fracture by 30-70%, depending on the drug used and adherence to the treatment regime (Papaioannou et al., 2010). The effect on nonvertebral fractures is lower and varies by fracture site. A list of pharmacologic options is provided in Table 6. The choice of a drug remains somewhat patient specific, but there are a number of first-line agents approved for treatment of osteoporosis in postmenopausal women at high risk of fracture including alendronate, risedronate, zoledronic acid, denosumab, raloxifene, hormone therapy, and teriparatide. As shown in Table 6, all of these agents have been shown to reduce vertebral fractures, and the majority also reduce hip and nonvertebral fractures. Each of these agents differs in dose, route of administration, and dosing frequency. For menopausal women requiring treatment for osteoporosis who also have vasomotor symptoms, hormone therapy can be used as a first-line treatment (Papaioannou et al., 2010). For men who require treatment for osteoporosis, alendronate, risedronate, zoledronic acid, and denosumab have been approved for use as first-line therapies. Testosterone is not recommended for the treatment of osteoporosis in men (Papaioannou et al., 2010) as it has not been shown to reduce the risk of fracture; however, it may be appropriately considered for those men with low bone mass or other symptoms related to androgen deficiency.

**Table 6:** Pharmacologic therapies

First-line therapies with evidence for fracture	Vertebral	Hip	Nonvertebral
prevention in postmenopausal women^a^	fracture	fracture	fracture^b^
Denosumab (60 mg SC twice yearly)	**✓**	**✓**	**✓**
Alendronate (10 mg PO daily or 70 mg PO weekly)	**✓**	**✓**	**✓**
Risedronate (5 mg PO daily, 35 mg PO weekly (regular tablet or delayed-release tablet), 75 mg PO monthly duet or 150 mg PO monthly)	**✓**	**✓**	**✓**
Zoledronic acid (5 mg IV yearly)	**✓**	**✓**	**✓**
Teriparatide (20 mcg SC daily)	**✓**	-	**✓**
Raloxifene (60 mg PO daily)	**✓**	-	-
Estrogen (hormone therapy)^c^	**✓**	**✓**	**✓**

a Check marks indicate a grade A recommendation for women. For men requiring treatment, alendronate, risedronate, and zoledronic acid can be used as first-line therapies for prevention of fractures (grade D).

b In clinical trials, nonvertebral fractures are a composite endpoint including hip, femur, pelvis, tibia, humerus, radius, and clavicle.

c Hormone therapy (estrogen) can be used as first-line therapy in women with menopausal symptoms. Adapted from Osteoporosis Canada (http://www.osteoporosis.ca/multimedia/FractureRiskTool/index.html#/Options).

Question: How do I select the appropriate medication and convey the importance of adhering to therapy to my patient?

*Answer*: Selection of an appropriate osteoporosis medication for a patient requires individualized consideration of patient preferences, commitment, and barriers to adherence to therapy depending on the patient's medical history, lifestyle factors, as well as the side effect profile of the drug being considered. Adherence to the treatment prescribed has been shown to greatly impact patient outcomes. Of interest, it is noted that physicians overestimate patient adherence to osteoporosis medications (69% of physicians estimated their patients to be adherent 80% of the time) whereas pharmacy claims showed that 49% were adherent after 1 year regardless of medication class or physician specialty (Copher et al., 2010). In a recent study of oral bisphosphonate adherence, after 1 year of prescribed therapy, adherence was 63% and declined to only 12% after 9 years (Burden et al., 2012). From an examination of U.S. claims databases it has been suggested that once adherence drops below 50%, fracture risk is increased, similar to not taking the medication at all (Siris et al., 2006). Patient reasons for nonadherence can be related to side effects, concerns about long-term risks with therapy, dissatisfaction with the medication, or practical inconveniences with drug administration (e.g., dosing, fasting requirement, and avoidance of co-administration with calcium with oral bisphosphonates; Carr, Thompson, & Cooper, 2006). In another study comparing a once weekly oral treatment to a once every 6 months injection treatment, after 1 year of treatment, the nonadherence rate was 46% lower on the injection treatment (Freemantle et al., 2012). Moreover, after the second year of these treatments the pattern of adherence continued and more than 90% of the patients preferred the every 6-month injections to the once weekly oral treatment (Freemantle et al., 2012). In a review, Papaioannou, Kennedy, Dolovich, Lau, and Adachi (2007) indicated that simplifying the medication administration regimen and minimizing adverse events would be key to increasing adherence. In addition, these investigators suggest regular monitoring with clinical and BMD reassessments, as well as ensuring the patient is provided with adequate instruction, educational material, and an opportunity to receive practitioner feedback and support.

**NP tip**

Those that are at high risk for fracture have the greatest benefit from medication therapy. For those in the moderate risk category, a discussion of fracture prevention, risk-to-benefit ratio of treatment, and education to involve patients in the decision process is essential. Those patients in the low-risk group require the triad of calcium, vitamin D, and weight-bearing exercise, which is foundational and also required in all risk categories.

### Case resolution

Mrs. X is at a moderate risk but has fallen twice in the last month. Taking the previous falls into consideration and the fact that she is fearful she may fall again, you recommend she contemplate medication treatment. Taking into account she has a history of GERD, as you discuss the potential risk of esophagitis related to oral bisphosphonates, you also provide her with parenteral options to consider, which include intravenously administered zoledronic acid or subcutaneously administered denosumab.

In view of this patient's 10-year fracture risk score, you provide her with educational material on osteoporosis including recommendations for calcium and vitamin D intake, exercise program options, as well as refer her to a fall prevention program. You book a follow-up appointment for 4 weeks from now to discuss further management and review pharmacotherapy options.

### Discussion

This article reviewed key clinical questions and answers relevant to NPs regarding the screening, assessment, and treatment of osteoporosis in a Canadian general practice setting. The case study highlighted the essential role that an NP can play in conducting a thorough update of the patient's history. Without specifically asking about bone health issues her moderate risk status for fragility fracture could have been missed. The NP has been shown to have a significant role in the screening of at-risk patients as demonstrated in the Kaiser Healthy Bones program in the United States where the use of DXA increased 263% with patient care managed by NPs from 2002 to 2007 (Greene & Dell, 2010). Moreover, NP managed patient care has been shown to increase use of medication for osteoporosis by 153% (Greene & Dell, 2010), demonstrating improved patient management. This care provided by NPs resulted in a 38% reduction in fractures from 2002 to 2007 (Greene & Dell, 2010).

### Conclusions

There is a considerable care gap in the assessment and treatment of men and women with osteoporosis in Canada. NPs have a unique opportunity to identify, risk stratify, and initiate treatment in patients at increased risk for osteoporosis and fracture, which can ultimately improve bone health in the aging Canadian population.
